# Na,K-ATPase Isozymes in Colorectal Cancer and Liver Metastases

**DOI:** 10.3389/fphys.2016.00009

**Published:** 2016-01-29

**Authors:** Marc Baker Bechmann, Deborah Rotoli, Manuel Morales, María del Carmen Maeso, María del Pino García, Julio Ávila, Ali Mobasheri, Pablo Martín-Vasallo

**Affiliations:** ^1^Laboratorio de Biología del Desarrollo, UD de Bioquímica y Biología Molecular and Centro de Investigaciones Biomédicas de Canarias, Universidad de La LagunaSanta Cruz de Tenerife, Spain; ^2^Institute of Endocrinology and Experimental Oncology, National Research CouncilNaples, Italy; ^3^Service of Medical Oncology, University Hospital Nuestra Señora de CandelariaSanta Cruz de Tenerife, Spain; ^4^Medical Oncology, Hospiten HospitalsSanta Cruz de Tenerife, Spain; ^5^Service of Pathology, University Hospital Nuestra Señora de CandelariaSanta Cruz de Tenerife, Spain; ^6^Department of Pathology, Hospiten HospitalsSanta Cruz de Tenerife, Spain; ^7^Department of Veterinary Preclinical Sciences, Faculty of Health and Medical Sciences, University of SurreyGuildford, UK; ^8^Faculty of Applied Medical Sciences, Center of Excellence in Genomic Medicine Research, King Fahd Medical Research Center, King AbdulAziz UniversityJeddah, Saudi Arabia

**Keywords:** Na/K-ATPase isozymes, sodium pump isozymes, colorectal cancer, colorectal cancer liver metastases, Na/K-ATPase isoforms colorectal cancer immunohistochemistry

## Abstract

The goal of this study was to define Na,K-ATPase α and β subunit isoform expression and isozyme composition in colorectal cancer cells and liver metastases. The α1, α3, and β1 isoforms were the most highly expressed in tumor cells and metastases; in the plasma membrane of non-neoplastic cells and mainly in a cytoplasmic location in tumor cells. α1β1 and α3β1 isozymes found in tumor and metastatic cells exhibit the highest and lowest Na^+^ affinity respectively and the highest K^+^ affinity. Mesenchymal cell isozymes possess an intermediate Na^+^ affinity and a low K^+^ affinity. In cancer, these ions are likely to favor optimal conditions for the function of nuclear enzymes involved in mitosis, especially a high intra-nuclear K^+^ concentration. A major and striking finding of this study was that in liver, metastasized CRC cells express the α3β1 isozyme. Thus, the α3β1 isozyme could potentially serve as a novel exploratory biomarker of CRC metastatic cells in liver.

## Introduction

Colorectal cancer (CRC) is one of the major causes of neoplasia-related morbidity and mortality, representing the second major cause of disease incidence among females and the third among males (Jemal et al., 2011). In the western world, CRC is the 4th leading cause of death (Ferlay et al., 2010). Metastatic CRC cells can invade, populate and flourish in a new niche and ultimately cause organ dysfunction and death. CRC spawns metastases in liver, lungs, bone marrow and brain (Chiang and Massague, 2008). However, it is the liver where CRC cells metastasize most frequently (Hess et al., 2006). The first line treatment for CRC involves surgery and adjuvant oxaliplatin based chemotherapy. A common side effect of this treatment strategy is oxaliplatin-induced peripheral neuropathy (Pachman et al., 2014).

Previous research from our group has led to the identification of several genes, which were shown to be significantly up-, or down- regulated in peripheral white cells (PWCs) of CRC patients, due to oxaliplatin-based chemotherapy (Morales et al., 2014). Interestingly, one of the differentially expressed genes was the isoform α3 of the *Na,K-ATPase*; mRNA levels of *Na,K-ATPase* α3 subunit were down-regulated 2.6-fold. Moreover, an alteration in the intracellular location of Na,K-ATPase α3 isoform has been reported in human CRC tumor cells vs. normal colon (Sakai et al., 2004). Additionally, other laboratories have shown differential expression in cells, altered subcellular localization and down regulation of the β subunit of the Na^+^/K^+^-ATPase in carcinoma cells (Rajasekaran et al., 1999, 2001a,b, 2010).

Na,K-ATPase is an integral protein in the plasma membrane of all animal cells that transports three sodium ions out and two potassium ions into the cell, against electrochemical gradient (Skou, 1957; Jorgensen et al., 2003). This activity is necessary for the regulation of the cellular ionic homeostasis and maintaining the electrochemical gradient required for ion channel function and secondary active transport (Mobasheri et al., 2000). Recently, additional functions for the Na,K-ATPase in the cell have been proposed, as a signal transducer and transcription activator (Aizman et al., 2001; Miyakawa-Naito et al., 2003; Harwood and Yaqoob, 2005; Yuan et al., 2005; Zhang et al., 2006) affecting cell proliferation (Abramowitz et al., 2003), cell motility (Barwe et al., 2005), and apoptosis (Wang and Yu, 2005). Besides this, the Na,K-ATPase is the receptor of cardiotonic glycosides. It is functionally composed of catalytic α (100–112 kDa) and regulatory β (45–55 kDa) subunit and an optional γ (6.5–10 kDa) subunit belonging to the FXYD family of proteins (Mercer et al., 1993).

Na,K-ATPase is expressed as several isozymes. Four different isoforms of the α subunit have been found in humans (Blanco, 2005). The α1 isoform (*ATP1A1* gene) is expressed almost in all tissues. Isoform α2 (*ATP1A2* gene) is the predominant isoform in skeletal muscle (Hundal et al., 1992), brain (astrocytes) (McGrail et al., 1991), heart (Zahler et al., 1992), and adipose tissue (Lytton et al., 1985). The α3 isoform (*ATP1A3* gene) is primarily found in the brain (neurons) (Hieber et al., 1991; McGrail et al., 1991) and isoform α4 (*ATP1A4* gene) is only expressed in testis (Woo et al., 2000). In reference to the β subunit, three different isoforms have been identified: β1 (*ATP1B1* gene), β2 (*ATP1B2* gene) and β3 (*ATP1B3* gene). While β1 has a generalized expression in almost all tissues and cells, the expression of the other β isoforms are more restricted to certain tissues and cells. The β2 isoform is found in skeletal muscle (Lavoie et al., 1997), pineal gland (Shyjan et al., 1990), and nervous tissues (Peng et al., 1997), whereas β3 is present in testis, retina, liver, and lung (Malik et al., 1996; Zahler et al., 1996; Arystarkhova and Sweadner, 1997; Martin-Vasallo et al., 2000). The expression pattern of the Na,K-ATPase subunit-isoforms is subjected to developmental and hormonal regulation and can be altered during disease (Book et al., 1994; Charlemagne et al., 1994; Charlemagne and Swynghedauw, 1995; Ewart and Klip, 1995; Zahler et al., 1996).

The purpose of this study was to determine the cellular and subcellular localization of the α and β subunit isoforms of Na,K-ATPase in CRC and its liver metastasis using a panel of well-characterized isoform-specific antibodies. The primary hypothesis of this study was that metastatic cancer cells possess a unique expression phenotype of Na,K-ATPase isozymes, similar to that of CRC cells.

## Materials and methods

### Tissue samples

The Ethics Committee of the Universidad de La Laguna (ULL) and Ethical Committee of the Hospital Universitario Nuestra Señora de Candelaria (HUNSC) approved this study. All patients signed an informed-consent document for diagnosis and research on tissue specimen before being enrolled in the project. All the study subjects were treated with FOLFOX CT: day 1 oxaliplatin 100 mg/m^2^ iv over 2 h; leucovorin calcium 400 mg/m^2^ iv over 2 h; followed by 5-fluorouracil 400 mg/m^2^ iv bolus and by 5-fluorouracil 2400 mg/m^2^ iv over 46 h; every 14 days. Paraffin-embedded tissue samples and clinical data were obtained from 15 patients (7 males, 8 females) and 1 control male from the reference medical areas of HUNSC.

### Antibodies

Table 1 shows antibodies and references used in this study. Secondary antibodies used were goat anti-rabbit IgG or goat anti-mouse IgG. Biotinylated secondary antibody was used for immunohistochemistry (IHC), whereas secondary antibodies targeted with specific fluorochromes were used for immunofluorescence (IF).

**Table 1:** **Antibodies used in this study. α1(620) (Sztul et al., 1987), α3 (Pietrini et al., 1992), α3 (XVIF9-G10) (Arystarkhova and Sweadner, 1996), SpETβ1 and SpETβ2 (Gonzalez-Martinez et al., 1994)**.

**Antibody**	**Target**	**Host**	**Type**	**Dilution**	**Source**
α1(620)	α1-isoform^*^	R	P	1:1000	M. J. Kashgarian
α3	α3-isoform^*^	R	P	1:600	M. Caplan
α3 (XVIF9-G10)	α3-isoform^*^	M	Mc	1:5	Arystarkhova and Sweadner
SpETβ1	β1-isoform^*^	R	P	1:600	P. Martin-Vasallo
SpETβ2	β2-isoform^*^	R	P	1:600	P. Martin-Vasallo
Anti-proliferating cell antigen (Anti-PCNA)	PCNA	M	Mc	1:100	Boehringer Mannheim
Anti-rabbit IgG (H+L), biotin conjugated (2°)	Rabbit-IgG	G	P	1:300	Pierce
Anti-rabbit IgG (whole molecule), FITC-conjugated (2°)	Rabbit-IgG	G	P	1:200	Sigma
Anti-mouse IgG, DyLight®650-conjugated (2°)	Mouse-IgG	G	P	1:100	Abcam

R, rabbit; G, goat; M, mouse; Mc, monoclonal; P, polyclonal.

* subunit-isoforms of the Na,K-ATPase, 2°: secondary antibody.

### Immunohistochemistry

Five-micron thick paraffin embedded tissue sections were deparaffinized in xylene and hydrated in graded series of alcohol baths. Heat mediated antigen retrieval was performed in an autoclave at 120°C for 10 min in sodium citrate buffer pH 6.0 before commencing the IHC staining protocol. To remove endogenous peroxidase activity, sections were incubated with 3% H_2_O_2_ in methanol for 15 min at room temperature. Non-specific sites were blocked with 5% Fetal Bovine Serum (FBS), 0.3% Triton-X-100 in Tris-buffered saline (TBS) for 1 h at room temperature. Endogen biotin was blocked with the Avidin/Biotin Vector Blocking Kit (Vector Laboratories Inc., #SP-2001, Burlingame, CA 94010, USA) according to the manufacturer's instructions. Primary antibodies (see Table 1) were incubated O/N at 4°C. Slices were then incubated for 2 h at 37°C with biotin-conjugated secondary antibodies (see Table 1). Antibodies for IHC were diluted in TBS, 5% FBS, 0.1% Triton. To amplify the specific antibody staining, ABC complex (Pierce, Thermo Fisher Scientific Inc., #32020, Waltham, MA, USA) was applied to the sections, prepared according to manufacturer's instruction and incubated for 1 h at room temperature. 3,3′-diaminobenzidine (DAB) Substrate Concentrate (Bethyl Laboratories Inc., #.IHC-101F, Montgomery, Texas, USA) was used to visualize immunoperoxidase activity. Slides were counterstained with Harris Hematoxylin solution DC (Panreac, #256991.1610 Barcelona, Spain) to visualize cell nuclei. Samples were mounted with Eukitt (Panreac, #253681, Barcelona, Spain) and optical light microscope (Olympus BX50, Tokyo, Japan) was used to visualize IHC staining results. Images were acquired using the Olympus DP70 camera and the DP controller software 2.1.1.183 (Copyright 2001–2004 Olympus Corporation). Negative control experiments were carried out by following the procedure stated above but without incubating with primary antibody.

### Double immunofluorescence simultaneous staining

As with the IHC samples, tissue sections for IF staining were paraffin embedded. After deparaffinization, hydration and heat-induced epitope retrieval procedure (as described above for the IHC staining), slides were incubated with 5%BSA, 0.3%Triton-X-100 in TBS to block non-specific sites. Then tissue sections were incubated simultaneously with a mixture of two distinct primary antibodies (e.g., rabbit against human target 1 and mouse against human target 2) overnight at 4°C. Slices were then incubated for 1 h at room temperature in dark with a mixture of two secondary antibodies (see Table 1) conjugated to two different fluorochromes (i.e., FITC-conjugated against rabbit-Sigma and DyLight®650-conjugated against mouse-Abcam). Antibodies for IF were diluted in TBS, 1% bovine serum albumin (BSA), 0.1% Triton. Slides were mounted with ProLong®Diamond Anti-fade Mountant with DAPI (Molecular Probes by Life Technologies, #P36962, Eugene, Oregon, USA) to visualize cell nuclei. Slides were acquired and analyzed using Olympus confocal microscope (Olympus FV1000, Tokyo, Japan) and the software FV10-ASW1.3; Lasers: Diode 405 nm, Argon multiline 458/488/514, HeNe 633 nm. Images were acquired by sequential scan (first sequence Diode and HeNe, second sequence Argon) to avoid overlapping of channels. Image resolution 1024 × 1024. Objective lens: 60X/1.35 NA oil Plan-Apochromat. Negative control experiments were carried out by following the same immunohistochemical procedure but with the primary antibody omitted.

### Image analysis and scoring

Samples were evaluated by two independent observers who were blinded to the clinical data. Scores were graded as absent (−), moderate (+) or strong (+++) for any specific kind of cell. These cut-offs were established by consensus of all investigators. For all tumors this grading was applied to three different patterns of Na,K-ATPase α and β subunit isoform staining in tumor cells: staining of the plasma membrane; staining of the nuclear envelope and staining of the cytoplasm.

Final results were computed as the product of staining intensities. In cases where scorings differed, the observers re-evaluated samples to consensus. All samples were analyzed and scored.

## Results

### Na,K-ATPase α1 and α3 isoform expression in CRC

**Figure 1:**
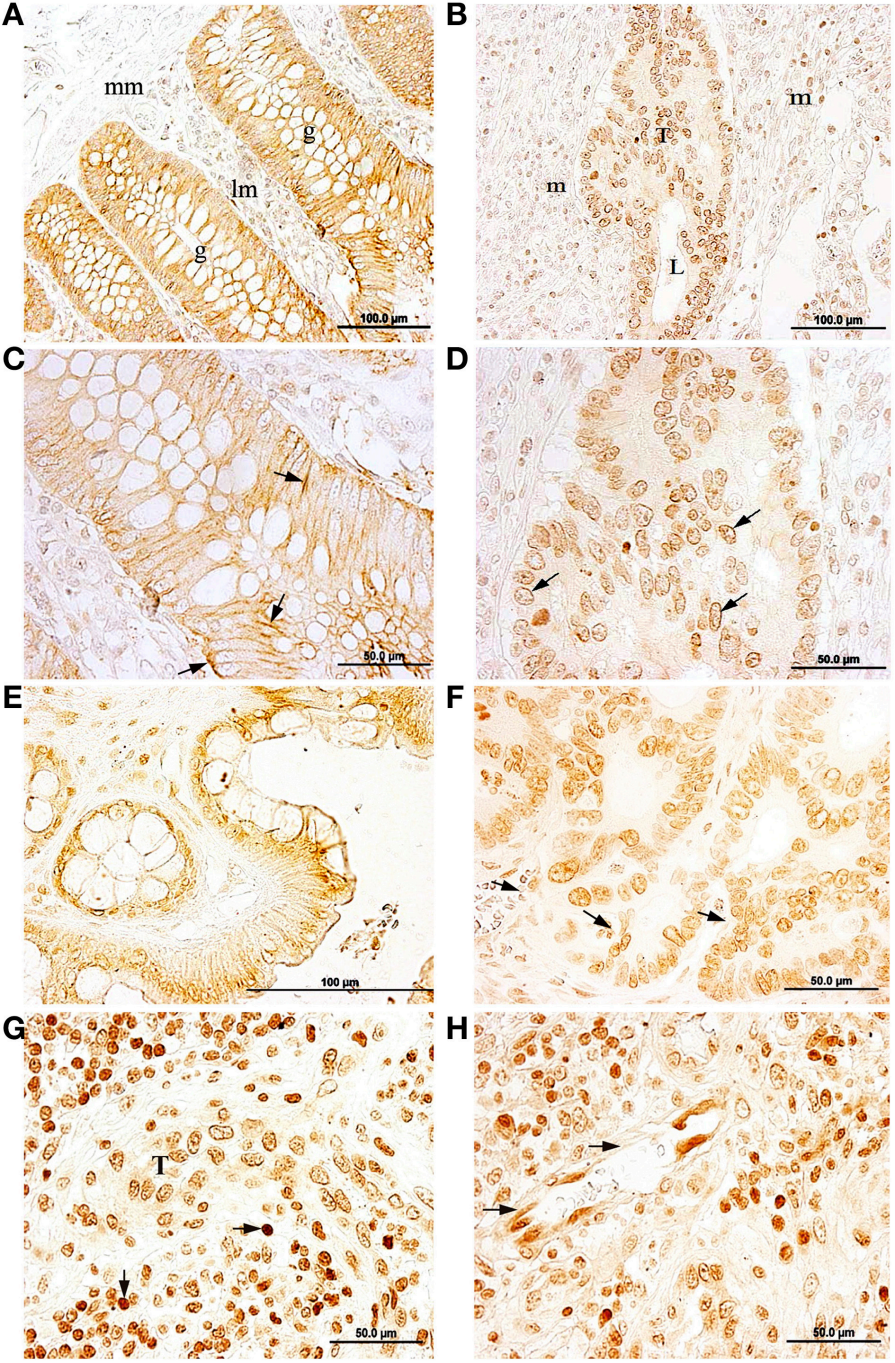
**Immunolocalization of the Na,K-ATPase α1 and α3 subunit isoforms in normal colon and colorectal cancer (CRC) (A,C)** α1 immunostaining in baso-lateral side of polarized epithelial cells in healthy colonic *mucosae*, black arrow. g, colonic gland (Lieberkühn crypts); mm, *muscularis mucosae*; lm, *lamina propia mucosae*. (**B,D)** peri-nuclear labeling for the α1 isoform in tumor cells. T, tumor; L, lumina; m, mesenchymal tissue. **(E)** α3 immunostaining in plasma membrane of healthy colon epithelial tissue. **(F)** α3 expressed peri-nuclearly in CRC tumor (arrows). **(G)** α3 immunostaining in tumor cells (T) surrounded by immune cells (arrows). **(H)** Endothelial cells expressing the α3 isoform in a peri-nuclear location (arrows).

In healthy colon tissue (Figures 1A,C), the α1 isoform was detected at the basolateral side of the plasma membrane of epithelial cells lining the colonic *mucosae* of Lieberkühn Crypts and in discrete stromal cells in the connective tissue surrounding the crypts. In turn, α1 isoform was mainly detected in a peri-nuclear location in tumor cells (Figures 1B,D). Mesenchymal cells from the stromal tissue surrounding the tumor also exhibited positive immunostaining (Figures 1B,D).

The Na,K-ATPase α3 isoform was detected on epithelial cells lining the colonic crypts and on cells from the *lamina propria* in healthy colon (Figure 1E). The α3 isoform was mainly detected in or near the plasma membrane of epithelial cells and in the cytoplasm of positively stained cells in the stroma. In CRC tumor samples, the α3 isoform was mainly located in a peri-nuclear location in CRC tumor cells, while in the plasma membrane of these cells staining was negative (Figure 1F). Stromal cells surrounding the tumor were also α3-positive. Immunolabeling for the α3 isoform was also detected in microvascular endothelial cells (Figure 1H) and in cells from the inflammatory reaction associated with CRC within the stroma (Figure 1G), where the α3 isoform showed an intense and specific peri-nuclear labeling.

### Na,K-ATPase β1 isoform expression in CRC

**Figure 2:**
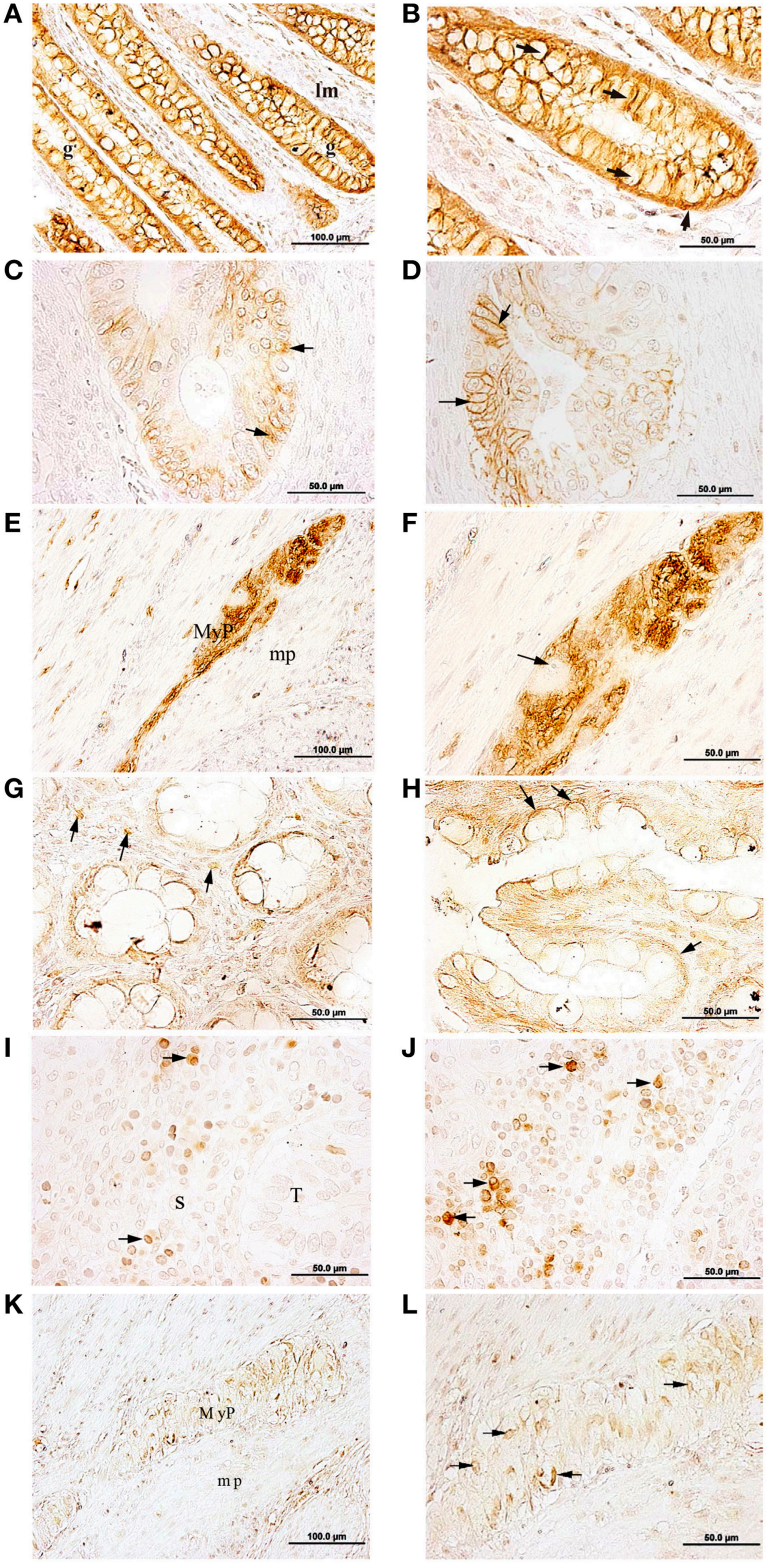
**Immunolocalization of the Na,K-ATPase β1 and β2 isoforms in normal colon and CRC**. **(A,B)** β1 is mainly located at the baso-lateral side of polarized epithelial cells from the normal colonic *mucosae* (arrows in **B**). **(C)** β1 is located in the cytosol of tumor cells (arrows). **(D)** β1 is located at the cell membrane of tumor cells (arrows). **(E)** Cells from the myenteric plexus (MyP) at the *muscularis propia* (mp) express β1. **(F)** β1 isoform is mainly located in or near the cytoplasmic membrane of the neurons and/or glia; a neuron nucleus with visible nucleolus is indicated using an arrow. g, colonic gland (Lieberkühn crypts); lm: *lamina propia mucosae*. **(G)** Selected cells from the connective tissue of the *mucosae* express the β2 isoform (arrows). **(H)** β2 is mainly located at the baso-lateral side of polarized epithelial cells from the normal colon *mucosae* (arrows). **(I)** β2 was not detected in tumor cells from adenocarcinomatous glands (T); however, some stroma (S) cells express β2 (arrows). **(J)** Possible leukocytes of the stroma expressing β2 (arrows). **(K)** Cells from the myenteric plexus (MyP) at the *muscularis propia* (mp) express β2. **(L)** β2 isoform is mainly located at the soma of glia cells (arrows).

The β1 isoform of Na,K-ATPase was detected in epithelial cells from the normal colonic *mucosae* (Figure 2A). There was a high positive staining at the baso-lateral side of polarized epithelial cells that line the colonic Lieberkühn crypts (Figure 2B).

In CRC tumors, the β1 isoform presented a less defined expression pattern. This isoform was detected in some tumor cells, but the location was not well-defined, and was seen in several subcellular locations within the tumor. In some tumor cells β1 staining was peri-nuclear (Figure 2C), while in others a peripheral location was observed (Figure 2C).

In addition, in healthy tissue, β1 was detected in cells of the myenteric plexus, also known as the Auerbach's plexus, within the muscular tissue of the *muscularis propia* (Figure 2E). In the cells from the plexus, Na,K-ATPase β1 isoform was detected in or near the cytoplasmic membrane of axons and dendrites of neurons and glial cells (Figure 2F).

### Na,K-ATPase β2 isoform expression in CRC

The β2 isoform was detected at the baso-lateral side of polarized epithelial cells from the normal colonic *mucosae* and in selected fibroblastic and immune cells from the *lamina propria* (Figure 2G) and (Figure 2H). In colon adenocarcinoma cells, the β2 isoform was not detected (Figures 2I,J). However, some immune cells located in the stromal tissue surrounding the tumor, were β2 positive while others remained negative (Figures 2I,J). In immunopositive cells, β2 staining was detected peri-nuclearly and also throughout the cytoplasm. Immunostaining intensity for the β2 isoform varied from strong to weak across cells in this region. In myenteric plexus (Figure 2K) the β2 isoform was detected in the soma of neural cells (Figure 2L).

### Co-expression of Na,K-ATpase α3 and β1 isoforms and PCNA

**Figure 3:**
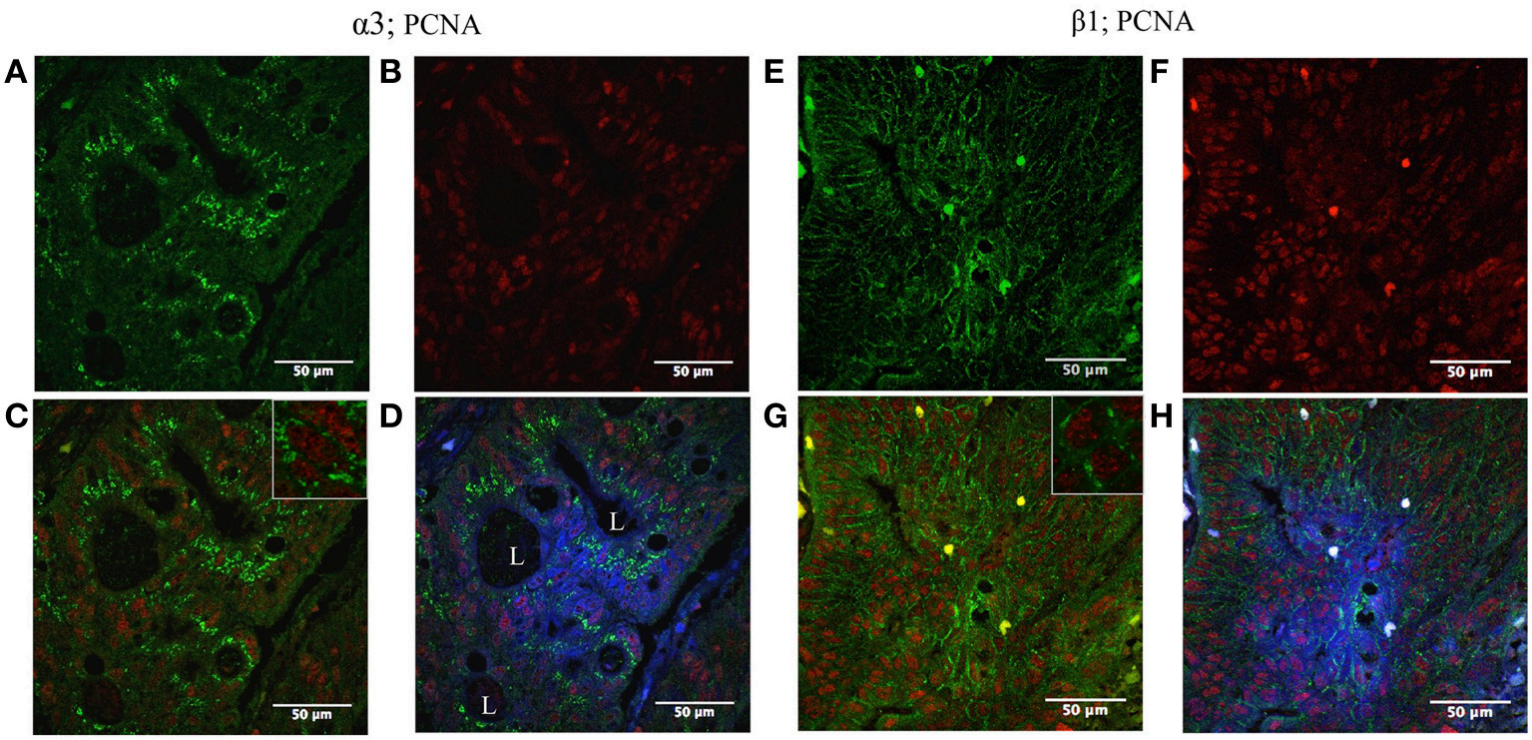
**Double immunofluorescence localization of Na,K-ATPase α3 and β1 isoform and proliferating cell nuclear antigen (PCNA) in CRC**. Left panel: **(A)** α3 isoform is expressed in colon tumor cells (green). **(B)** High numbers of tumor cells express PCNA (red). **(C)** The tumor cells express both PCNA and the α3 isoform (blue and green merged). **(D)** α3 isoform is mainly located internally at the cytoplasm; blue (DAPI), red (PCNA), and green (α3 isoform) merged image. L, lumina. Right panel: **(E)** β1 isoform is expressed in colonic tumor cells (green). **(F)** High number of tumor cells expresses PCNA (red). **(G)** The tumor cells express both PCNA and β1 isoform (red and green merged). **(H)** Blue (DAPI), red (PCNA), and green (β1 isoform) merged image.

In CRC, some cells from adenocarcinomatous glands showed positive staining to both PCNA (nuclei) and Na,K-ATPase α3 isoform (cytoplasm) (Figure 3), and PCNA and β1 isoform (plasma membrane) (Figure 3).

### Na,K-ATPase α1 and α3 isoform expression in CRC metastases in liver

**Figure 4:**
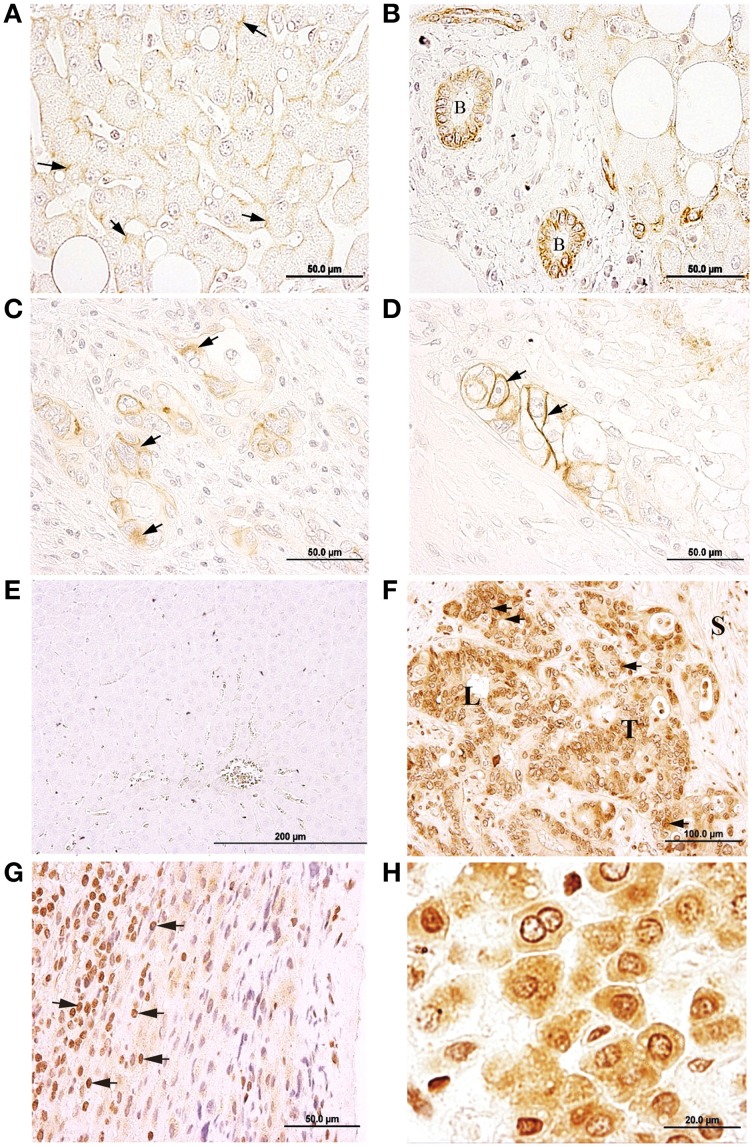
**Immunolocalization of the Na,K-ATPase α1 and α3 isoforms in normal liver and liver metastasis**. **(A)** α1 isoform is located at the plasma membrane of the hepatocytes (arrows). **(B)** Bile duct epithelial cells (B) express α1 isoform, mainly at the baso-lateral side of cell membrane. **(C)** α1 isoform is located at the cytosol of metastatic tumor cells (arrows). **(D)** α1 is present at a peripheral position in metastatic tumor cells (arrows). **(E)** Negative immunolocalization of the α3 isoform in normal liver tissue. **(F)** α3 isoform is mainly located at a peri-nuclear position but also detected all-over the cytoplasm. **(G)** Inflammatory reaction established at the outermost part of the liver; α3 IHC-positive cells may correspond to cells from the immune system (arrows), such as leukocytes. **(H)** In normal liver tissue from a metastasized liver, hepatocytes express α3 in peri-nuclear and cytoplasmic locations.

In healthy liver tissue, hepatocytes were immunopositive for α1, in the plasma membrane (Figure 4A). Bile ducts cells were also α1 positive (Figure 4B), and the staining was detected mainly at the baso-lateral side of the plasma membrane. In metastases, the Na,K-ATPase α1 isoform was detected in cytoplasm and in the cytoplasmic membrane of cells of metastatic tumor niches (Figures 4C,D).

In normal healthy liver, Na,K-ATPase α3 isoform was not detected in any cell types (Figure 4E). However, in metastatic tumor cells within the liver, the α3 isoform was detected (Figure 4F; Supplementary Figure S1D) in a peri-nuclear location and spread across the cytoplasm. Staining intensity varied among cells, from strong to weak labeling. In addition to tumor cells, this isoform was also detected in immune cells located at the outermost part of the liver (Figure 4G). In apparently healthy liver tissue surrounding metastases, Na,K-ATPase α3 isoform was detected peri-nuclearly and also throughout the cytoplasm of hepatocytes (Figure 4H).

### Na,K-ATPase β1 and β2 isoforms expression in metastasis

**Figure 5:**
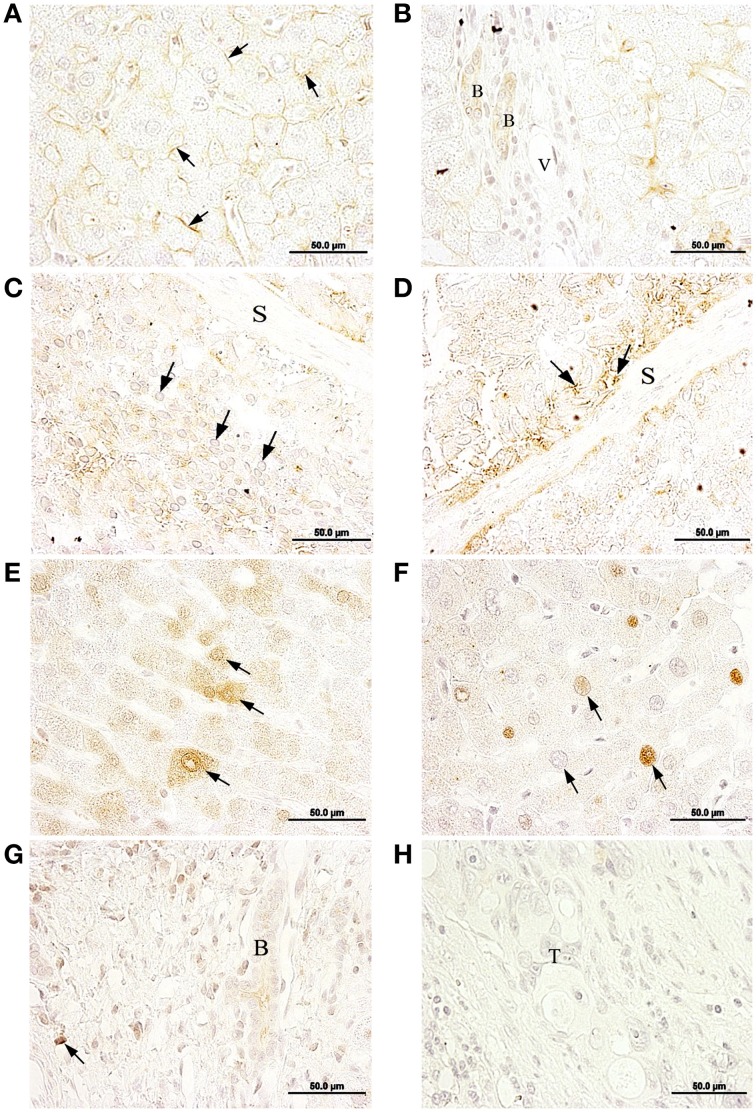
**Immunolocalization of the Na,K-ATPase β1 and β2 isoforms in normal liver tissue and liver metastasis**. **(A)** β1 isoform is mainly located at the cytoplasmic membrane of hepatocytes (arrows) from normal liver. **(B)** Bile duct epithelial cells (B) express β1, V, venule. **(C)** Nuclei of metastatic tumor cells (arrows). **(D)** β1 IHC-positive staining at necrotic tissue is signaled with arrows; S, septum. **(E)** β2 is differentially expressed at the cytoplasm of neighboring hepatocytes (arrows) of normal liver. **(F)** Hepatocytes with different degrees of β2 immunostaining at a peri-nuclear position in neighboring hepatocytes (arrows) of normal liver. **(G)** In normal liver, a bile duct (B) and cells from the lining connective tissue (arrow) express β2. **(H)** Metastatic tumor cells (T) do not express β2.

In metastasized liver, the β1 isoform was detected at the plasma membrane of hepatocytes (Figure 5A), bile ducts epithelial cells (Figure 5B) and peri-nuclearly and/or in the cytoplasm of cells in disorganized and necrotic tissue (Figures 5C,D; Supplementary Figure S1C).

In healthy liver tissue, the β2 isoform was detected in the cytoplasm of some cells and in peri-nuclear locations in others (Figures 5E,F), with variable staining intensities among cells ranging from strong to weak. A weak but specific signal for the β2 isoform was also detected in bile ducts cells at the portal triads (Figure 5G). However, the β2 isoform was not detected in metastatic tumor cell niches (Figure 5H).

We were unable to detect the α2 and β3 subunit isoforms as their expression levels were probably below the threshold of the ABC amplified immunohistochemical detection technique employed in this study.

### Na,K-ATPase α3 and β1 isoforms coexpression in metastasis

**Figure 6:**
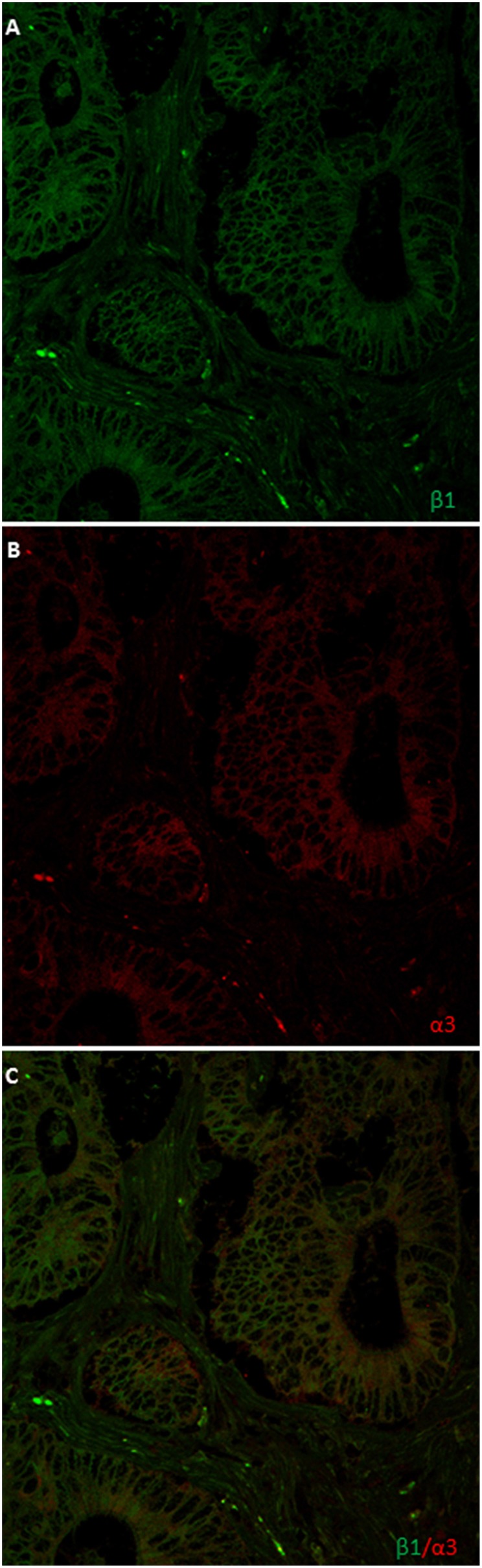
**Double immunofluorescence localization of Na,K-ATPase α3 and β1 isoform in liver metastasis**. **(A)** α3 isoform is located at a peri-nuclear position and all-over the cytoplasm. **(B)** β1 isoform is mainly located at the plasma membrane of metastases cells and in nuclear envelope. **(C)** Merge.

In order to further confirm the co-expression of the α3 and β1 subunits isoforms in the same metastatic cells in the liver, using a different and monoclonal antibody, we performed confocal microscopy co-localization experiments. Image analysis and scoring was done using same procedure as in all other cases and stated in the Materials and Methods section. As shown in Figure 6 and in Supplementary Figure S1, both of them co-localize in a number of metastatic cells in percentages ranging from + to +++, depending on the sample and on the area within the same sample. Most of them showed further more than 2/3 of total metastatic cells.

## Discussion

In this study we explored the cellular and subcellular localization of the α and β subunit isoforms of Na,K-ATPase in CRC and its liver metastases. The aim of this work was to test the hypothesis that metastatic cancer cells possess a unique expression phenotype of Na,K-ATPase isozymes, which may be similar to that of CRC cells. Table 2 summarizes the cell-specific Na,K-ATPase subunit-isoforms expression and Table 3 highlights the possible cell-specific Na,K-ATPase isozymes present in healthy colon, colorectal cancer, healthy liver and metastasized liver.

**Table 2:** **Cell-specific Na,K-ATPase subunit-isoform expression in normal colon, colorectal cancer, normal liver, and metastasized liver**.

	**α1**	**α3**	**β1**	**β2**
**Normal colon**				
Epithelial cells (*Mucosae*)	**+++**	**+++**	**+++**	**+**
Mesenchymal cells (S*ubmucosae*)	**+**	**+**	**+**	**+**
Smooth muscle cells (*Muscularis mucosae*)	**?**	**?**	**?**	**?**
Neurons (Myenteric plexus)	**?**	**+**	**+++**	**?**
Glia cells (Myenteric plexus)	**?**	**?**	**+++**	**+++**
Smooth muscle cells (*Muscularis propia*)	**?**	**+**	**−**	**+**
**Colorectal cancer**				
Tumor cells	**+++**	**+++**	**+++**	**−**
Mesenchymal cells (not immune system cells)	**+**	**+++**	**−**	**+**
Immune system cells	**?**	**+++**	**?**	**?**
Endothelial cells	**?**	**+++**	**−**	**?**
Epithelial cells (*Mucosae*)	+++^*^	**+++**	**+++**	**−**
**Normal liver**				
Hepatocytes	**+++**	**−**	**+++**	**+**
Ephitelial cells (bile duct)	**+++**	**−**	**+**	**+**
Endothelial cells	**−**	**−**	**−**	**−**
Mesenchymal cells (connective tissue)	**−**	**−**	**−**	**+**
**Metastasized liver**				
Tumor cells	**+++**	**+++**	**+**	**−**
Hepatocytes	**?**	**+**	**?**	−^*^
Epithelial cells (Bile duct)	**+++**	**?**	**?**	**?**
Mesenchymal cells (connective tissue)	**−**	**+**	**−**	**−**
Immune system cells	**−**	**+++**	**?**	**?**

+++, indicates a high staining level; +, indicates low staining level; −, indicates no staining detected; and ?, indicates indeterminate staining, according to our observations.

* Data not shown.

**Table 3:** **Possible cell-specific Na, K-ATPase isozymes present in normal colon, colorectal cancer, normal liver, and metastasized liver**.

	**α1β1**	**α1β2**	**α3β1**	**α3β2**
**NORMAL COLON**				
Epithelial cells (*Mucosae*)	+++	+	+++	+
Mesenchymal cells (*Submucosae*)	+	+	+	+
Smooth muscle cells (*Muscularis mucosae*)	?	?	?	?
Neurons (Myenteric plexus)	?	?	+++	?
Glia cells (Myenteric plexus)	?	?	?	?
Smooth muscle cells (*Muscularis propia*)	–	?	–	+++
**COLORECTAL CANCER**				
Tumor cells	+++	–	+++	–
Mesenchymal cells (Not immune system cells)	–	+	–	+++
Immune system cells	?	?	?	?
Endothelial cells	–	?	–	?
Epithelial cells (*Mucosae*)	+++	–	+++	–
**NORMAL LIVER**				
Hepatocytes	+++	+	–	–
Ephitelial cells (Bile duct)	+++	+++	–	–
Endothelial cells	–	–	–	–
Mesenchymal cells (Connective tissue)	–	–	–	–
**METASTIZED LIVER**				
Tumor cells	+++	–	+++	–
Hepatocytes	?	–	?	–
Epithelial cells (Bile duct)	?	?	?	?
Mesenchymal cells (Connective tissue)	–	–	–	–
Immune system cells	–	–	?	?

*+++, Indicates a possible high level of the isozyme; +, indicates a possible low presence of the isozyme; –, indicates no possibility; and ?, indicates indeterminate staining, according to our observations*.

Based on the results presented we propose that the predominating isozymes in tumor cells from the colon and metastases in the liver are α1β1 and α3β1. The α3β1 isozyme of Na,K-ATPase is only present in liver metastases but not in healthy liver, thus, the α3β1 isozyme could serve as a novel exploratory biomarker of CRC metastatic cells in liver. Further studies should be carried out to test the utility of this observation.

Subunits of Na,K-ATPase have the ability to form functional isoenzymes by a promiscuous association of α and β isoforms to confer significantly different kinetic and biological properties. The apparent affinities for cations and ouabain have been determined by expressing recombinant enzymes in heterologous systems. Affinities of human isozymes expressed in *Xenopus laevis* oocytes are α1β1>α2β1>α3β1 for Na^+^ and α3β1=α1β1>α1β3>α1β2>α2β1>α3β3>α3β2>α2β3>α2β2 for K^+^ (Blanco and Mercer, 1998; Crambert et al., 2000). Tumor cells and metastatic cells have isozymes with highest and lowest Na^+^ affinity and surrounding mesenchymal cells possess isozymes with medium range affinity. Regarding K^+^ affinity, tumor and metastasis cells possess Na,K-ATPase isozymes of high K^+^ affinity and mesenchymal cells low K^+^ affinity. These are the isozyme combinations that permit an optimal performance of the enzymes involved in protein synthesis and transfer of phosphor groups (Glynn, 1985) processes both involved in carcinogenesis.

Another objective of this study was to correlate the mitotic index related to the expression of isoforms by co-localization of those along with PCNA, the clamp subunit of DNA polymerase δ marker of cell proliferation (Kubben et al., 1994; Bleau et al., 2014) and carried out further analysis by confocal microscopy. Figure 3 shows cells expressing the α3 and β1 isoforms in CRC tumor cells, in which no correlation was seen between sodium pump isoforms and PCNA protein expression, that is, high expression of PCNA can be found in cells with either, high or low, expression level of α3 or β1 and vice versa.

Hideki Sakai and co-workers (Sakai et al., 2004) used western blotting to demonstrate a decrease in α1 isoform expression in CRC and, inversely, an increase in the α3 isoform compared to the accompanying healthy *mucosae*. In addition they did not observe a significant expression level of Na,K-ATPase α2 isoform either in the CRC or in the accompanying healthy *mucosae*. Our study, confirms their observations. In addition, in recent studies of hepatocellular carcinoma, a significantly higher α3 level expression was shown in western blots compared to the accompanying non-tumor tissues, whereas no significant increases in expression of α1 and α2 proteins was observed (Shibuya et al., 2010).

Recently, it has been suggested that the cellular distribution and expression of Na,K-ATPase α3 isoform affects the anti-proliferative effects of oleandrin, a cardiac glycoside that inhibits the Na,K-ATPase (Yang et al., 2014). These authors demonstrated that healthy, as opposed to neoplastic colonic and lung tissues, exhibit different distributions of the α3 isoform. While the α3 isoform was predominantly located in the cytoplasmic membrane in healthy colon and lung, the distribution of this isoform was shifted to a predominantly peri-nuclear location in tumors. These observations have been corroborated by our laboratory. Furthermore, our results showed a subcellular location shift for the α1 isoform, which was mainly located at the basolateral side of the plasma membrane of healthy colonic epithelial cells, shifting to a peri-nuclear position in CRC tumor cells.

The α1 and α3 subunit isoforms were detected in all cells lining the colonic crypts. These isoforms were not only expressed in epithelial cells in healthy colon *mucosae*, but they were also detected in mesenchymal cells from the *lamina propria*. The α3 isoform presents a high expression level in neurons of the central nervous system (Hieber et al., 1991; McGrail et al., 1991). The present study shows specific staining for α3 in neurons from myenteric plexus.

Regarding the β subunit, it has been reported that expression of both the β1 and β2 mRNAs were decreased in renal, lung and hepatocellular carcinomas (Akopyanz et al., 1991), and that expression levels of the corresponding proteins was decreased in human clear cell renal cell carcinoma (Rajasekaran et al., 1999) and bladder carcinoma (Espineda et al., 2004). Previous work from our laboratory (Avila et al., 1997) reported, by western blot technique, that gastric and colon adenocarcinoma showed opposite patterns of β1 isoform expression. While gastric adenocarcinomas showed lower expression levels of β1 than the healthy tissue, colonic adenocarcinomas showed higher expression of this isoform compared to healthy surrounding tissue. In addition, the β2 isoform was neither detected in healthy colon, nor in stomach adenocarcinomas. In the present immunohistochemical study, we detected the β1 and β2 isoforms at the baso-lateral side of the plasma membrane in the healthy colon *mucosae*, but only β1 was found in CRC samples. Which, in a certain manner, resembles our previous findings in Na,K-ATPase in dog and rat prostate cancer where we found a downregulation and a reduced expression of sodium pump (Mobasheri et al., 2000, 2003a,b,c).

Na,K-ATPase β1 and β2 isoforms were detected in the myenteric plexus of healthy colon tissue. It is well-established that the β2 isoform of Na,K-ATPase, is an adhesion molecule on glia (AMOG) (Antonicek et al., 1987; Gloor et al., 1990).

In tumor samples, the β1 isoform presented a less defined pattern of expression. This isoform was detected in some tumor cells but not all, also the subcellular location differed among cells within a given adenocarcinomatous area, while some tumor cells where immunopositive for β1 at the cytoplasmic membrane location (Figure 2D) other cells presented immunostaining in a peri-nuclear position (Figure 2C).

Research by Rajasekaran and colleagues reported that Na,K-ATPase β subunit is required for epithelial polarization, suppression of invasion, and cell motility (Rajasekaran et al., 2001b), not only presence of Na,K-ATPase in the cell membrane but also Na,K-ATPase activity was important to form proper tight junctions, desmosomes, and induction of polarity in epithelial cells (Rajasekaran et al., 2001b). Further studies suggested that the transcription factor Snail might be repressing the β1 isoform and E-cadherin expression in carcinomas, associating these events to epithelial-mesenchymal transition (EMT) (Espineda et al., 2004).

Taken together, these studies and our results indicate that the level of expression and the location of the β subunit in epithelial cells are important for maintaining their well-differentiated phenotype, which disappears during cancer progression. Research published by our group on sodium pump isoform expression levels in stem cells has confirmed that adipose-derived mesenchymal stem cells express all known Na,K-ATPase isoforms, but some of these genes are turned off along differentiation (Acosta et al., 2011). In CRC cells or its metastases in liver we have never seen expression of all isoforms, rather, we have detected the expression signatures specified in Table 2.

Regarding liver metastases, to our knowledge, there have not been any reported studies in reference to Na,K-ATPase isoforms in liver metastasis. In this study the α1 isoform was detected in metastatic tumor cell niches within the liver, exhibiting a cytoplasmic subcellular localization in some cells and a membrane localization in others. The α3 isoform, however, was mainly detected at a peri-nuclear location and was more diffusely expressed across the cytoplasm of tumor metastatic cells. The healthy hepatic tissue presented the α1 isoform at the cytoplasmic membrane where it may establish the functional heterodimer with a β1 isoform and/or β2 isoform. Our observations of the subcellular localization of the α1 isoform are consistent with the first reported immunolocalization of this subunit in hepatocytes of healthy liver tissue (Sztul et al., 1987). However, in healthy liver tissue, the α3 isoform was not detected.

The β2 isoform was detected both at a more peri-nuclear position in some hepatocytes and throughout the cytoplasm in others, but not at the cytoplasmic membrane. The reason for this is unclear at present. It is possible that β2 isoform performs other *moonlighting* protein functions in hepatocytes. In metastasized liver, we detected the β1 isoform in disordered and semi-necrotic tumor tissue. However, β2 isoform was not detected in liver metastases. This may be related to the fact that these metastatic cells arise from CRC tumor cells, which did neither express β2 isoform or at very insignificant levels. Interestingly, apparently in healthy hepatic tissue surrounding the metastatic zone, the hepatocytes expressed the α3 isoform, a phenotype not detected in healthy hepatocytes from non-CRC patient according to our results. It might be possible that the CRC and the FOLFOX-CT affecting this patient could be influencing these hepatocytes driving them to express other genes, *ATP1A3* in this case.

The high levels of peri-nuclear and cytoplasmic α3 isoform in liver metastatic cells is potentially indicative of other *moonlighting* functions of this isoform besides ion transport (Jeffery, 2014; Magpusao et al., 2015; Min et al., 2015). In addition, the α3β1 isozyme may have utility as a novel exploratory biomarker for metastases cells. However, further studies need to be performed in order to confirm both, the moonlighting and biomarker assessments.

## Author contributions

Conceived and designed the study and experiments: MM and PV. Patients were selected by: MM. Performed the experiments: MB, DR, MdCM, MG, and JÁ. Analyzed and discussed the data and discussed the written manuscript: All authors. Wrote the manuscript: MB, DR, AM, and PV. Constructed the figures and tables: MB, DR, and JÁ.

### Conflict of interest statement

The authors declare that the research was conducted in the absence of any commercial or financial relationships that could be construed as a potential conflict of interest.

## References

[B1] Abramowitz J. Dai C. Hirschi K. K. Dmitrieva R. I. Doris P. A. Liu L.. (2003). Ouabain- and marinobufagenin-induced proliferation of human umbilical vein smooth muscle cells and a rat vascular smooth muscle cell line, A7r5. Circulation 108, 3048–3053. 10.1161/01.CIR.0000101919.00548.86 14638550

[B2] Acosta E. Avila J. Mobasheri A. Martin-Vasallo P. (2011). Na+, K+-ATPase genes are down-regulated during adipose stem cell differentiation. Front. Biosci.(Elite Ed). 3, 1229–1240. 10.2741/326 21622129

[B3] Aizman O. Uhlen P. Lal M. Brismar H. Aperia A. (2001). Ouabain, a steroid hormone that signals with slow calcium oscillations. Proc. Natl. Acad. Sci. U.S.A. 98, 13420–13424. 10.1073/pnas.221315298 11687608 PMC60886

[B4] Akopyanz N. S. Broude N. E. Bekman E. P. Marzen E. O. Sverdlov E. D. (1991). Tissue-specific expression of Na,K-ATPase beta-subunit. Does beta 2 expression correlate with tumorigenesis? FEBS Lett. 289, 8–10. 10.1016/0014-5793(91)80896-B 1654277

[B5] Antonicek H. Persohn E. Schachner M. (1987). Biochemical and functional characterization of a novel neuron-glia adhesion molecule that is involved in neuronal migration. J. Cell Biol. 104, 1587–1595. 10.1083/jcb.104.6.1587 2438288 PMC2114497

[B6] Arystarkhova E. Sweadner K. J. (1996). Isoform-specific monoclonal antibodies to Na,K-ATPase alpha subunits. Evidence for a tissue-specific post-translational modification of the alpha subunit. J. Biol. Chem. 271, 23407–23417. 10.1074/jbc.271.38.23407 8798546

[B7] Arystarkhova E. Sweadner K. J. (1997). Tissue-specific expression of the Na,K-ATPase beta3 subunit. The presence of beta3 in lung and liver addresses the problem of the missing subunit. J. Biol. Chem. 272, 22405–22408. 10.1074/jbc.272.36.22405 9278390

[B8] Avila J. Lecuona E. Morales M. Soriano A. Alonso T. Martin-Vasallo P. (1997). Opposite expression pattern of the human Na,K-ATPase beta 1 isoform in stomach and colon adenocarcinomas. Ann. N. Y. Acad. Sci. 834, 653–655. 10.1111/j.1749-6632.1997.tb52341.x 9405883

[B9] Barwe S. P. Anilkumar G. Moon S. Y. Zheng Y. Whitelegge J. P. Rajasekaran S. A.. (2005). Novel role for Na,K-ATPase in phosphatidylinositol 3-kinase signaling and suppression of cell motility. Mol. Biol. Cell. 16, 1082–1094. 10.1091/mbc.E04-05-0427 15616195 PMC551475

[B10] Blanco G. (2005). Na,K-ATPase subunit heterogeneity as a mechanism for tissue-specific ion regulation. Semin. Nephrol. 25, 292–303. 10.1016/j.semnephrol.2005.03.004 16139684

[B11] Blanco G. Mercer R. W. (1998). Isozymes of the Na-K-ATPase: heterogeneity in structure, diversity in function. Am. J. Physiol. 275, F633–F650. 9815123 10.1152/ajprenal.1998.275.5.F633

[B12] Bleau A. M. Agliano A. Larzabal L. de Aberasturi A. L. Calvo A. (2014). Metastatic dormancy: a complex network between cancer stem cells and their microenvironment. Histol. Histopathol. 29, 1499–1510. 10.14670/HH-29.1499 24887025

[B13] Book C. B. Wilson R. P. Ng Y. C. (1994). Cardiac hypertrophy in the ferret increases expression of the Na(+)-K(+)-ATPase alpha 1- but not alpha 3-isoform. Am. J. Physiol. 266, H1221–H1227. 8160826 10.1152/ajpheart.1994.266.3.H1221

[B14] Charlemagne D. Orlowski J. Oliviero P. Rannou F. Sainte B. C. Swynghedauw B.. (1994). Alteration of Na,K-ATPase subunit mRNA and protein levels in hypertrophied rat heart. J. Biol. Chem. 269, 1541–1547. 8288620

[B15] Charlemagne D. Swynghedauw B. (1995). Myocardial phenotypic changes in Na+, K+ ATPase in left ventricular hypertrophy: pharmacological consequences. Eur. Heart J. 16(Suppl. C), 20–23. 10.1093/eurheartj/16.suppl_C.20 7556267

[B16] Chiang A. C. Massague J. (2008). Molecular basis of metastasis. N. Engl. J. Med. 359, 2814–2823. 10.1056/NEJMra0805239 19109576 PMC4189180

[B17] Crambert G. Hasler U. Beggah A. T. Yu C. Modyanov N. N. Horisberger J. D.. (2000). Transport and pharmacological properties of nine different human Na, K-ATPase isozymes. J. Biol. Chem. 275, 1976–1986. 10.1074/jbc.275.3.1976 10636900

[B18] Espineda C. E. Chang J. H. Twiss J. Rajasekaran S. A. Rajasekaran A. K. (2004). Repression of Na,K-ATPase beta1-subunit by the transcription factor snail in carcinoma. Mol. Biol. Cell. 15, 1364–1373. 10.1091/mbc.E03-09-0646 14699059 PMC363145

[B19] Ewart H. S. Klip A. (1995). Hormonal regulation of the Na(+)-K(+)-ATPase: mechanisms underlying rapid and sustained changes in pump activity. Am. J. Physiol. 269, C295–C311. 7653511 10.1152/ajpcell.1995.269.2.C295

[B20] Ferlay J. Shin H. R. Bray F. Forman D. Mathers C. Parkin D. M. (2010). Estimates of worldwide burden of cancer in 2008: GLOBOCAN 2008. Int. J. Cancer 127, 2893–2917. 10.1002/ijc.25516 21351269

[B21] Gloor S. Antonicek H. Sweadner K. J. Pagliusi S. Frank R. Moos M.. (1990). The adhesion molecule on glia (AMOG) is a homologue of the beta subunit of the Na,K-ATPase. J. Cell Biol. 110, 165–174. 10.1083/jcb.110.1.165 1688561 PMC2115981

[B22] Glynn I. M. (1985). The Na-K transporting adenosine triphosphatase, in The enzymes of Biological Membranes, 2nd Edn., eds Martonosi A. N. (New York, NY: Plenum Press), 35–114.

[B23] Gonzalez-Martinez L. M. Avila J. Marti E. Lecuona E. Martin-Vasallo P. (1994). Expression of the beta-subunit isoforms of the Na,K-ATPase in rat embryo tissues, inner ear and choroid plexus. Biol. Cell. 81, 215–222. 10.1016/0248-4900(94)90003-5 7696974

[B24] Harwood S. Yaqoob M. M. (2005). Ouabain-induced cell signaling. Front. Biosci. 10, 2011–2017. 10.2741/1676 15970473

[B25] Hess K. R. Varadhachary G. R. Taylor S. H. Wei W. Raber M. N. Lenzi R.. (2006). Metastatic patterns in adenocarcinoma. Cancer 106, 1624–1633. 10.1002/cncr.21778 16518827

[B26] Hieber V. Siegel G. J. Fink D. J. Beaty M. W. Mata M. (1991). Differential distribution of (Na, K)-ATPase alpha isoforms in the central nervous system. Cell Mol. Neurobiol. 11, 253–262. 10.1007/BF00769038 1851465 PMC11567476

[B27] Hundal H. S. Marette A. Mitsumoto Y. Ramlal T. Blostein R. Klip A. (1992). Insulin induces translocation of the alpha 2 and beta 1 subunits of the Na+/K(+)-ATPase from intracellular compartments to the plasma membrane in mammalian skeletal muscle. J. Biol. Chem. 267, 5040–5043. 1312081

[B28] Jeffery C. J. (2014). An introduction to protein moonlighting. Biochem. Soc. Trans. 42, 1679–1683. 10.1042/BST20140226 25399589

[B29] Jemal A. Bray F. Center M. M. Ferlay J. Ward E. Forman D. (2011). Global cancer statistics. CA Cancer J. Clin. 61, 69–90. 10.3322/caac.20107 21296855

[B30] Jorgensen P. L. Hakansson K. O. Karlish S. J. (2003). Structure and mechanism of Na,K-ATPase: functional sites and their interactions. Annu. Rev. Physiol. 65, 817–849. 10.1146/annurev.physiol.65.092101.142558 12524462

[B31] Kubben F. J. Peeters-Haesevoets A. Engels L. G. Baeten C. G. Schutte B. Arends J. W.. (1994). Proliferating cell nuclear antigen (PCNA): a new marker to study human colonic cell proliferation. Gut 35, 530–535. 10.1136/gut.35.4.530 7909785 PMC1374804

[B32] Lavoie L. Levenson R. Martin-Vasallo P. Klip A. (1997). The molar ratios of alpha and beta subunits of the Na+-K+-ATPase differ in distinct subcellular membranes from rat skeletal muscle. Biochemistry 36, 7726–7732. 10.1021/bi970109s 9201913

[B33] Lytton J. Lin J. C. Guidotti G. (1985). Identification of two molecular forms of (Na+,K+)-ATPase in rat adipocytes. Relation to insulin stimulation of the enzyme. J. Biol. Chem. 260, 1177–1184. 2981837

[B34] Magpusao A. N. Omolloh G. Johnson J. Gascon J. Peczuh M. W. Fenteany G. (2015). Cardiac glycoside activities link Na(+)/K(+) ATPase ion-transport to breast cancer cell migration via correlative SAR. ACS Chem. Biol. 10, 561–569. 10.1021/cb500665r 25334087 PMC4340362

[B35] Malik N. Canfield V. A. Beckers M. C. Gros P. Levenson R. (1996). Identification of the mammalian Na,K-ATPase 3 subunit. J. Biol. Chem. 271, 22754–22758. 10.1074/jbc.271.37.22754 8798450

[B36] Martin-Vasallo P. Wetzel R. K. Garcia-Segura L. M. Molina-Holgado E. Arystarkhova E. Sweadner K. J. (2000). Oligodendrocytes in brain and optic nerve express the beta3 subunit isoform of Na,K-ATPase. Glia 31, 206–218. 10.1002/1098-1136(200009)31:3<206::AID-GLIA20>3.0.CO;2-1 10941147

[B37] McGrail K. M. Phillips J. M. Sweadner K. J. (1991). Immunofluorescent localization of three Na,K-ATPase isozymes in the rat central nervous system: both neurons and glia can express more than one Na,K-ATPase. J. Neurosci. 11, 381–391. 1846906 10.1523/JNEUROSCI.11-02-00381.1991 PMC6575210

[B38] Mercer R. W. Biemesderfer D. Bliss D. P. Jr. Collins J. H. Forbush B. III. (1993). Molecular cloning and immunological characterization of the gamma polypeptide, a small protein associated with the Na,K-ATPase. J. Cell Biol. 121, 579–586. 10.1083/jcb.121.3.579 8387529 PMC2119561

[B39] Min K. W. Lee S. H. Baek S. J. (2015). Moonlighting proteins in cancer. Cancer Lett. 370, 108–116. 10.1016/j.canlet.2015.09.022 26499805 PMC4684795

[B40] Miyakawa-Naito A. Uhlen P. Lal M. Aizman O. Mikoshiba K. Brismar H.. (2003). Cell signaling microdomain with Na,K-ATPase and inositol 1,4,5-trisphosphate receptor generates calcium oscillations. J. Biol. Chem. 278, 50355–50361. 10.1074/jbc.M305378200 12947118

[B41] Mobasheri A. Avila J. Cozar-Castellano I. Brownleader M. D. Trevan M. Francis M. J.. (2000). Na+, K+-ATPase isozyme diversity; comparative biochemistry and physiological implications of novel functional interactions. Biosci. Rep. 20, 51–91. 10.1023/A:1005580332144 10965965

[B42] Mobasheri A. Evans I. Martin-Vasallo P. Foster C. S. (2003a). Expression and cellular localization of Na,K-ATPase isoforms in dog prostate in health and disease. Ann. N. Y. Acad. Sci. 986, 708–710. 10.1111/j.1749-6632.2003.tb07286.x 12763922

[B43] Mobasheri A. Fox R. Evans I. Cullingham F. Martin-Vasallo P. Foster C. S. (2003b). Epithelial Na, K-ATPase expression is down-regulated in canine prostate cancer; a possible consequence of metabolic transformation in the process of prostate malignancy. Cancer Cell Int. 3, 8. 10.1186/1475-2867-3-8 12848899 PMC194866

[B44] Mobasheri A. Pestov N. B. Papanicolaou S. Kajee R. Cozar-Castellano I. Avila J.. (2003c). Expression and cellular localization of Na,K-ATPase isoforms in the rat ventral prostate. BJU Int. 92, 793–802. 10.1046/j.1464-410X.2003.04460.x 14616469

[B45] Morales M. Avila J. Gonzalez-Fernandez R. Boronat L. Soriano M. L. Martin-Vasallo P. (2014). Differential transcriptome profile of peripheral white cells to identify biomarkers involved in oxaliplatin induced neuropathy. J. Pers. Med. 4, 282–296. 10.3390/jpm4020282 25563226 PMC4263976

[B46] Pachman D. R. Loprinzi C. L. Grothey A. Ta L. E. (2014). The search for treatments to reduce chemotherapy-induced peripheral neuropathy. J. Clin. Invest. 124, 72–74. 10.1172/JCI73908 24355918 PMC3871261

[B47] Peng L. Martin-Vasallo P. Sweadner K. J. (1997). Isoforms of Na,K-ATPase alpha and beta subunits in the rat cerebellum and in granule cell cultures. J. Neurosci. 17, 3488–3502. 9133374 10.1523/JNEUROSCI.17-10-03488.1997 PMC6573685

[B48] Pietrini G. Matteoli M. Banker G. Caplan M. J. (1992). Isoforms of the Na,K-ATPase are present in both axons and dendrites of hippocampal neurons in culture. Proc. Natl. Acad. Sci. U.S.A. 89, 8414–8418. 10.1073/pnas.89.18.8414 1326755 PMC49930

[B49] Rajasekaran S. A. Ball W. J. Jr. Bander N. H. Liu H. Pardee J. D. Rajasekaran A. K. (1999). Reduced expression of beta-subunit of Na,K-ATPase in human clear-cell renal cell carcinoma. J. Urol. 162, 574–580. 10.1016/S0022-5347(05)68629-6 10411090

[B50] Rajasekaran S. A. Huynh T. P. Wolle D. G. Espineda C. E. Inge L. J. Skay A.. (2010). Na,K-ATPase subunits as markers for epithelial-mesenchymal transition in cancer and fibrosis. Mol. Cancer Ther. 9, 1515–1524. 10.1158/1535-7163.MCT-09-0832 20501797 PMC2884047

[B51] Rajasekaran S. A. Palmer L. G. Moon S. Y. Peralta S. A. Apodaca G. L. Harper J. F.. (2001a). Na,K-ATPase activity is required for formation of tight junctions, desmosomes, and induction of polarity in epithelial cells. Mol. Biol. Cell. 12, 3717–3732. 10.1091/mbc.12.12.3717 11739775 PMC60750

[B52] Rajasekaran S. A. Palmer L. G. Quan K. Harper J. F. Ball W. J. Jr. Bander N. H.. (2001b). Na,K-ATPase beta-subunit is required for epithelial polarization, suppression of invasion, and cell motility. Mol. Biol. Cell 12, 279–295. 10.1091/mbc.12.2.279 11179415 PMC30943

[B53] Sakai H. Suzuki T. Maeda M. Takahashi Y. Horikawa N. Minamimura T.. (2004). Up-regulation of Na(+),K(+)-ATPase alpha 3-isoform and down-regulation of the alpha1-isoform in human colorectal cancer. FEBS Lett. 563, 151–154. 10.1016/S0014-5793(04)00292-3 15063740

[B54] Shibuya K. Fukuoka J. Fujii T. Shimoda E. Shimizu T. Sakai H.. (2010). Increase in ouabain-sensitive K+-ATPase activity in hepatocellular carcinoma by overexpression of Na+, K+-ATPase alpha 3-isoform. Eur. J. Pharmacol. 638, 42–46. 10.1016/j.ejphar.2010.04.029 20447393

[B55] Shyjan A. W. Cena V. Klein D. C. Levenson R. (1990). Differential expression and enzymatic properties of the Na+,K(+)-ATPase alpha 3 isoenzyme in rat pineal glands. Proc. Natl. Acad. Sci. U.S.A. 87, 1178–1182. 10.1073/pnas.87.3.1178 2153972 PMC53434

[B56] Skou J. C. (1957). The influence of some cations on an adenosine triphosphatase from peripheral nerves. Biochim. Biophys. Acta 23, 394–401. 10.1016/0006-3002(57)90343-8 13412736

[B57] Sztul E. S. Biemesderfer D. Caplan M. J. Kashgarian M. Boyer J. L. (1987). Localization of Na+,K+-ATPase alpha-subunit to the sinusoidal and lateral but not canalicular membranes of rat hepatocytes. J. Cell Biol. 104, 1239–1248. 10.1083/jcb.104.5.1239 3032985 PMC2114466

[B58] Wang X. Q. Yu S. P. (2005). Novel regulation of Na, K-ATPase by Src tyrosine kinases in cortical neurons. J. Neurochem. 93, 1515–1523. 10.1111/j.1471-4159.2005.03147.x 15935067

[B59] Woo A. L. James P. F. Lingrel J. B. (2000). Sperm motility is dependent on a unique isoform of the Na,K-ATPase. J. Biol. Chem. 275, 20693–20699. 10.1074/jbc.M002323200 10764792

[B60] Yang P. Cartwright C. Efuet E. Hamilton S. R. Wistuba I. I. Menter D.. (2014). Cellular location and expression of Na+, K+ -ATPase alpha subunits affect the anti-proliferative activity of oleandrin. Mol. Carcinog. 53, 253–263. 10.1002/mc.21968 23073998 PMC4442617

[B61] Yuan Z. Cai T. Tian J. Ivanov A. V. Giovannucci D. R. Xie Z. (2005). Na/K-ATPase tethers phospholipase C and IP3 receptor into a calcium-regulatory complex. Mol. Biol. Cell 16, 4034–4045. 10.1091/mbc.E05-04-0295 15975899 PMC1196317

[B62] Zahler R. Brines M. Kashgarian M. Benz E. J. Jr. Gilmore-Hebert M. (1992). The cardiac conduction system in the rat expresses the alpha 2 and alpha 3 isoforms of the Na+,K(+)-ATPase. Proc. Natl. Acad. Sci. U.S.A. 89, 99–103. 10.1073/pnas.89.1.99 1309618 PMC48183

[B63] Zahler R. Gilmore-Hebert M. Sun W. Benz E. J. (1996). Na, K-ATPase isoform gene expression in normal and hypertrophied dog heart. Basic Res. Cardiol. 91, 256–266. 10.1007/BF00788912 8831945

[B64] Zhang S. Malmersjo S. Li J. Ando H. Aizman O. Uhlen P.. (2006). Distinct role of the N-terminal tail of the Na,K-ATPase catalytic subunit as a signal transducer. J. Biol. Chem. 281, 21954–21962. 10.1074/jbc.M601578200 16723354

