# Supplementary material for: Na,K-ATPase Isozymes in Colorectal Cancer and Liver Metastases
Source: Front Physiol. 2016 Jan 29;7:9. doi: 10.3389/fphys.2016.00009 (PMC4731494; doi:10.3389/fphys.2016.00009)
Supplement: Supplementary file 1 [file Image1.PDF]

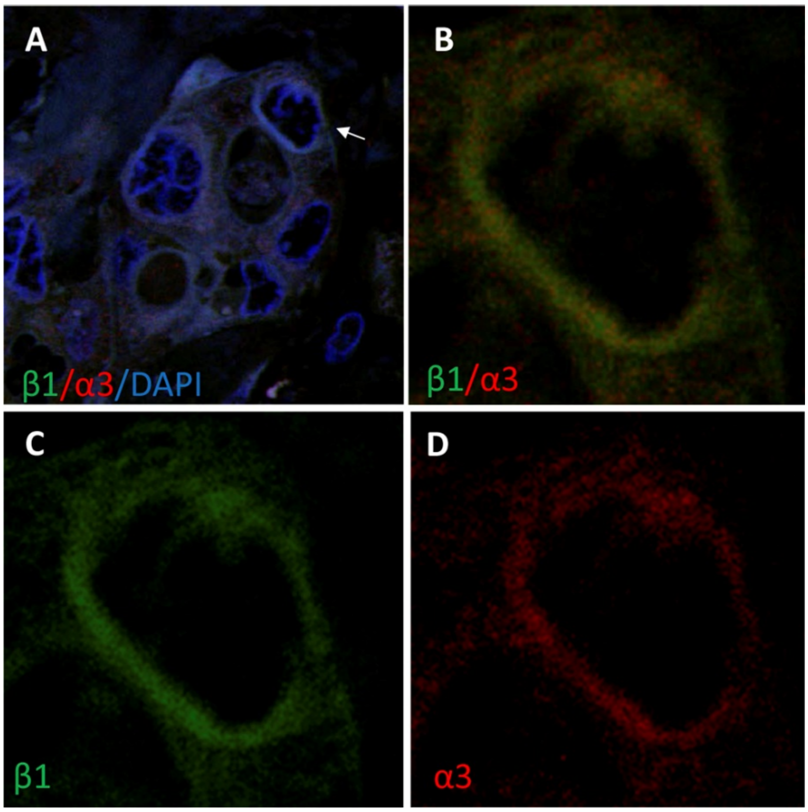

Legend of supplementary Figure S1:

Double immunofluorescence localization of Na,K-ATPase α3 (red) and β1(green) isoforms in liver metastasis. A) α3 and β1 isoforms co-localize at the nuclear envelope in malignant cells. B-D) Magnification of the malignant cell pointed in A; B) α3 and β1 merge. C) β1. D) α3.
